# Ninety‐Seven Percent of Trials Investigating Robotic Interventions in Physiotherapy Contained Abstract Spin: A Meta‐Research Review

**DOI:** 10.1002/cesm.70072

**Published:** 2026-02-12

**Authors:** Hilary Tier, Jana Verveer, David B. Anderson, Camila Quel De Oliveira, Nicci Bartley, Poonam Mehta, Rafael Z. Pinto, Arianne P. Verhagen, Alana B. McCambridge, Peter W. Stubbs

**Affiliations:** ^1^ Discipline of Physiotherapy, Graduate School of Health, Faculty of Health University of Technology Sydney Sydney New South Wales Australia; ^2^ Department of Rehabilitation, Physiotherapy Research and Sport, UMC Utrecht Brain Center Universiteit Utrecht Utrecht the Netherlands; ^3^ Sydney School of Health Sciences, Faculty of Medicine and Health The University of Sydney Sydney New South Wales Australia; ^4^ Discipline of Physiotherapy, School of Health Sciences Western Sydney University Sydney New South Wales Australia; ^5^ PoCoG, School of Psychology, Faculty of Science The University of Sydney Sydney New South Wales Australia; ^6^ AUT Person Centred Rehabilitation Research Centre, Faculty of Health and Environmental Sciences Auckland University of Technology Auckland New Zealand

**Keywords:** agreement, clinical trials, misreporting, misrepresentation, neurology, robotics, technology

## Abstract

**Background:**

Abstract spin involves misrepresenting or misreporting study findings in the abstract of an article. The abstract is the most easily accessible part of the article and may determine if an article is read, purchased or the findings are implemented into practice. Trials using new technologies, such as robotics, may be particularly vulnerable to spin due to the high costs associated with research and development.

**Objective:**

To identify and assess abstract spin in physiotherapy clinical trials investigating robotic interventions.

**Design:**

Meta‐research review.

**Methods:**

We searched the Physiotherapy Evidence Database (PEDro) in August 2024 for two‐armed clinical trials investigating robotic interventions compared to nonrobotic interventions, in any patient population. Article screening and data extraction were performed by two people independently. Quality assessment was performed using the PEDro scale with PEDro scores ≥ 6 deemed high quality. Abstract spin was assessed by two independent raters using a 7‐item checklist. Spin items were scored “present,” “not present” or “not applicable.” Data were presented as counts and percentages.

**Results:**

We included 160 trials, of which 95% were in neurological physiotherapy and 61% of trials were high quality. Almost all trials (97%) contained at least one item of spin. Most often abstracts failed to mention adverse events (90%) or overenthusiastically interpretated non‐significant (primary) outcomes (77%). One percent of abstracts clearly omitted negative primary outcomes, and 23% of abstracts recommended treatments without clinically important effects on the primary outcomes. These low spin percentages were due to many trials not reporting any negative finding and trials not providing a clinical recommendation in the abstract.

**Conclusion:**

Ninety‐seven percent of abstracts in trials investigating robotic interventions in physiotherapy contained spin. Academic journals should be conscious of the high prevalence of abstract spin in robotic trials and consider implementing stricter author guidelines or peer‐review practices to ensure abstracts truly reflect the study findings.

## 1 Background

Abstract spin is the misreporting, misrepresentation, and inappropriate extrapolation of a study's findings in an abstract, which omits or fails to faithfully reflect the methods or results and usually provides a more positive interpretation of the study [1, 2]. Spin is common in medical literature [3, 4] with increased attention to spin in recent years [5, 6, 7, 8, 9, 10, 11, 12, 13]. Although spin can occur throughout an article, it is particularly impactful in an abstract. The abstract is often the first part of an article that a reader has access to, readers may choose to read or purchase an article based on the abstract and some people may only read the abstract [14, 15]. Given this, abstracts that contain spin are more likely to have their full texts read and interventions potentially implemented. The importance of spin in an abstract was shown in a study of cancer trials [16]. Three‐hundred clinicians were randomized to read two abstracts, one with spin and the other without, and asked to rate the benefit of the treatment, rigorousness of the methods and interested in reading the full article [16]. Clinicians presented with the abstract with spin rated the treatment as more beneficial, reported methods as being more rigorous and were more likely to want to read the full text [16]. For these reasons, it is important that abstracts fairly reflect the findings of the study so that readers can make informed and appropriate decisions.

There are multiple reasons for abstract spin and findings framed more positively than they are. For academics, the pressure to publish is high with job performance indicators often linked to number of publications and impact factor of the journal of publication [17]. As research with positive results is easier and more likely to be published, academics may feel compelled to present results that are framed more positively [17]. In pharmaceutical research there are often large costs involved in manufacturing, prototyping and initial nonclinical and clinical testing. This means publishing an article with a positive abstract may be preferred to gain interest in the treatment and increase its uptake. For example, when assessing the whole article, pharmaceutical studies funded by for‐profit organizations are more likely to report positive conclusions, due to biased interpretation of study results [18]. When findings are misrepresented or misreported, consumers of research may use or advocate for treatments that don't provide the expected effect, are ineffective, or potentially harmful.

Many studies have investigated spin in biomedical literature and abstract spin has been investigated in multiple clinical areas including health and medical research [19], pharmacology [20], surgery [7, 21, 22, 23, 24, 25, 26], dentistry [27, 28, 29, 30, 31, 32, 33, 34, 35, 36], and physiotherapy [6, 37, 38, 39]. Abstract spin in these areas had considerable variability, ranging from 3% [34] to 98% [6] of included articles. One area that abstract spin has not been investigated is in interventions using robotics. Studies using robotic interventions are particularly vulnerable to abstract spin due to the high costs associated with research and development. The worldwide spending on rehabilitation robots is high, and individual units can cost as much as $500,000 (USD). Given this, there are large incentives to show that these are effective. Cochrane reviews on upper‐ [40] and lower‐limb [41] robotics in stroke populations against physiotherapy or usual care show moderate to high certainty evidence that posttreatment between group differences are only small to moderate for activity of daily living scores (standardized mean difference (SMD 0.31), arm function (SMD 0.32), arm strength (SMD 0.46), 6‐min walk distance (11 m), mean walking velocity (0.05 m/s), and independent walking (odds ratio 1.65). A review investigating only overground robotic exoskeletons showed very‐low certainty evidence for all outcomes [42]. Studies in people with acquired brain injury [43], mixed neurological conditions [44], spinal cord injury [45] and Parkinsons Disease [46] mainly showed low or very‐low certainty evidence, and the few comparisons with moderate to high certainty evidence showed small effect sizes. Given the small effect sizes and uncertain evidence, as well as the cost of the devices to hospitals and research institutions, there is a high risk that these studies may want to oversell the results to make an intervention appear more effective.

Given this, it is important to investigate spin in studies using robotic interventions as this has not yet been investigated, and there is a high possibility of abstract spin. For this article we investigated abstract spin using the 7‐item checklist developed by Nascimento et al. [6] informed by the widely referenced guidelines developed by Boutron [3]. In our protocol article, we provided expanded descriptions of each item from that checklist to reduce the ambiguity of scoring [47]. For the current study, we have chosen to use this checklist with updated definitions [47]. The primary aim of the current study was to assess the amount and type of abstract spin in physiotherapy robotics clinical trials indexed in PEDro. The secondary aims are to provide rater agreement in using this spin checklist and explore potential factors related to spin.

## 2 Methods

### 2.1 Design

This study has a meta‐research design using guidance from the Preferred Reporting Items for Systematic reviews and Meta‐Analyses (PRISMA) guidelines [48]. The protocol was published on October 19, 2020 [47].

### 2.2 Search Strategy

The search was performed in the Physiotherapy Evidence database (PEDro) and has been described previously [47]. PEDro indexes clinical trials, systematic reviews, and clinical practice guidelines within the scope (or future scope) of physiotherapy practice [49]. PEDro is updated using monthly searches of Medline, Embase, CINAHL, CENTRAL AMED, and PsycINFO and articles are included in the database regardless of registration status, journal, language or methodological quality [49, 50, 51]. Included articles were clinical trials indexed in PEDro. Searches were performed using the “clinical trials” filter, using terms searching the titles and abstracts related to robotics. These were “robot*’” “Exoskel*,” “Electro mechanic*,” “Electromechanic*,” “automat*,” “orthotic*,” “orthos*,” “*driven,” “computer aided” and “computer assist*.” The search was performed on August 2, 2024.

### 2.3 Selection Criteria

Included trials were two‐armed randomized trials written in English investigating a robotic intervention (alone or with another nonrobotic intervention) compared to a non‐robotic intervention. We excluded multi‐armed trials to ensure that the trials were as comparable as possible as trials with more arms would have more primary comparisons which may increase the likelihood of spin. We defined a robotic intervention as “a machine capable of carrying out a complex series of actions automatically” [47, 52, p. 2]. Some of examples of devices that are considered robotics are the Lokomat [53], Ekso‐GT [54], Morning Walk [55], Hybrid Assistive Limb [56], Bi‐Manu‐Track [57], InMotion‐1 and ‐2 (MIT‐MANUS) [58, 59], Armeo Spring [60], and Neuro‐Rehabilitation‐Robot (NeReBot) [61]. Some examples of interventions that are not considered robotics are the SaeboFlex [62], wearable exoskeleton stride management assist system (SMA) [63], Trunk stabilization training robot (3DBT‐33) [64], SMART Arm [65], Neurocom Pro‐Balance Master [66], Therasuit [67], LOCOBOT [68], Hunova [69], Motorized Ankle Stretcher [70], AnkleMotus [71], and experimental devices used to elicit stretch reflexes [72, 73, 74]. Trials could investigate any population, outcome or timepoint. Excluded studies were pseudo‐randomized studies, nonrandomized studies, cross‐over trials, cluster trials or studies that had robotic interventions in both study arms.

### 2.4 Study Selection

Screening of the title/abstract and full text for eligible trials were independently performed against the inclusion/exclusion criteria by two raters in Covidence. Disagreements were discussed until consensus was reached.

### 2.5 Data Extraction

All included trials underwent spin assessment and data extraction. Data were extracted by two authors independently using a custom‐made data extraction form in Excel (Version 16.70 (Build 23021201)). Disagreements in data extraction were resolved through discussion. Extracted data were the number of authors, publication year, patient population, geographical location, abstract length, number of randomized patients, number of primary outcomes and timepoints, funding and funding type, conflict of interest statement, and if a conflict of interest was reported, journal impact factor and trial preregistration.

### 2.6 Quality Assessment

Trial quality was rated using the PEDro quality assessment scale [75, 76]. The PEDro scale contains 11 items, of which 10 are scored. The first item relates to external validity and is not scored. Items 2–9 related to the internal validity with questions on randomization, concealment, baseline similarities, blinding (patient, therapist and assessor), loss to follow‐up and intention to treat analysis. Items 10 and 11 relate to statistical reporting and includes reporting of point estimates and variability and between‐group statistical comparisons. Scores are summed from 0 (*no items met*) to 10 (*all items met*). In our study, we used the ratings (from 0 to 10) provided on the PEDro website. These have been rated by two experienced raters and verified by a third rater.

### 2.7 Assessment of Spin

We used the spin‐item categories defined previously [6] with updated item descriptions [47] (Appendix 2). Item descriptions were expanded to reduce ambiguity of the initial descriptions [47]. The items were omission of primary outcomes (item 1), failing to report between‐group non‐significant primary outcomes (item 2), selectively reporting of positive results and omission of negative between‐group results of primary outcomes (item 3), focussing on statistically significant outcomes other than the primary (item 4), failing to mention adverse events of the intervention (item 5), overenthusiastically interpreting statistically nonsignificant primary outcomes results as effective (item 6), recommending a treatment without a clinically important effect on the primary outcome (item 7) [13, 41]. Items 1 and 5 were rated “Yes” or “No” with all other items rated “Yes,” “No” or “Not Relevant.”

Raters assessed if the abstract demonstrated spin in corroboration with the full text, with primary outcomes/timepoints being determined from an investigation of the full text. For spin item 1, the same primary outcomes/timepoints also needed to be mentioned in the abstract. If between group primary outcomes/timepoints were non‐significant (item 2) or negative (item 3) in the text, these needed to be presented in the abstract. When the full‐text did not provide between‐group differences, these were calculated using the mean, SD and number in each group using a calculator to determine the mean difference and 95% confidence intervals [77]. For item 4, the between‐group primary outcome/timepoint needed to be omitted in favor of a secondary outcome (that didn't need to be mentioned in the full‐text). For the measurement of adverse events, if stated in the abstract, these needed to align with the adverse events mentioned in the full‐text. For item 6, when nonsignificant between group differences of primary outcomes had been determined in the full‐text, abstracts could provide overenthusiastic reporting of within‐group differences or used statements implying significance, whether mentioned in the full‐text or not. For determination of the smallest clinically worthwhile effect (SCWE), we used the SCWE provided in the full‐text. When this was not provided, we used 15% of the scale range when a scale was used or a 15% difference between measures of the postintervention value of the intervention group for continuous measures.

If the abstract or full‐text of a trial did not nominate a primary outcome all outcomes were deemed primary. If the abstract or full‐text of a trial did not nominate a primary timepoint, all measurement timepoints were deemed primary. If the abstract or full‐text of the trial only measured one outcome or one timepoint, that outcome or timepoint was deemed primary.

Two authors independently rated the spin items using a custom‐made data extraction form in Qualtrics. If initial ratings did not agree, consensus was attained through discussion and, if required, through consultation with a third author.

### 2.8 Data Analysis

Data on included trial characteristics were tabulated using counts, percentages and means (standard deviation), where appropriate. The number of trials that used spin were tabulated for each spin item and Yes, No and Not relevant responses were summarized as counts and percentages.

*Agreement in spin ratings:* Agreement in spin ratings was compared between authors using Fleiss' kappa for items with “Yes” and “No” responses only (items 1 and 5) or “Yes,” “Not Yes” (No and Not Relevant) (items 2–4, 6 and 7) as per the protocol. Kappa values were interpreted as slight (0–0.20), minimal (0.21–0.39), weak (0.40–0.59), moderate (0.60–0.79), strong (0.80–0.90), and almost perfect (> 0.90) agreement [78]. Fleiss' *κ* values were tabulated as the *κ* value (95% Confidence intervals) with the percentage agreement.

*Exploratory analyses:* One‐way analysis of variance (ANOVA), unpaired *t*‐tests and linear regression analyses were performed to assess potential differences/relationships between independent variables and the amount of spin. For all tests, the amount of spin was the dependent variable (on a 0–7 scale). Independent variables assessed using one‐way ANOVA were continent (North America, Asia, Europe, Other), Funding reported (Yes, No, N/A [i.e., No funding section]), whether the trial declared a Conflict of Interest (COI) (Yes, No, N/A (No COI section)), whether the trial was linked to industry through COI or funding (Yes, No, unknown), journal 2‐year citations per document (from Scimago Journal and Country Rank) (no data and 0–1, 1–2, 2–5, > 5) and abstract word length (≤ 175, 176–225, 226–275, 276–325, ≥ 326). ANOVA results were presented as the *F*‐value with degrees of freedom and *p* value. If ANOVA were significant, post hoc *t*‐tests were performed to identify the differences between groups. Independent variables assessed using unpaired *t*‐tests were protocol registration (Yes, No), area of physiotherapy (Neurology, Other) and whether the primary outcome was defined (Yes, No). These were presented as mean differences (MD) and 95% confidence intervals (95% CI). Independent variables assessed using linear regression analyses were PEDro score (1–10) and year of publication. These were presented as unstandardized beta coefficients (unstandardized *β*) and 95% CI.

### 2.9 Deviations From the Protocol

We intended to choose a selection of 100 randomized clinical trials. However, as the number of trials was only 160, we decided to assess all trials. We intended to include quasi‐randomized trials but changed the inclusion criteria to only include randomized trials. This was to ensure the included trials were as rigorous as possible. The spin checklist was piloted by spin raters by screening five trials not included in the final selection. Item 7 required a SCWE to determine if authors used spin in their clinical interpretation of the findings. If a SCWE was reported for primary outcomes in the manuscript, this was used. Initially, when this was not reported in the primary manuscript, authors searched the available literature for the SCWE. However, in practice screening, 0/5 trials reported a SCWE, so we stopped searching for the SCWE in the literature as (1) it became too time‐consuming (i.e., One trial had 20+ primary outcomes), (2) the SCWE often did not exist for the outcome measures relating to many of the populations of interest, severity of condition and phase postinjury, and (3) there were often sometimes multiple (different) SCWE estimates. As such, we developed an arbitrary rule that the SCWE was 15% of the scale range when a scale was used or a 15% difference between measures of the postintervention value of the intervention group for continuous measures. In the protocol, we intended to compare spin of experienced and inexperienced raters. Data detailing inexperienced raters were presented elsewhere [79].

## 3 Results

### 3.1 Search Results

**Figure 1:**
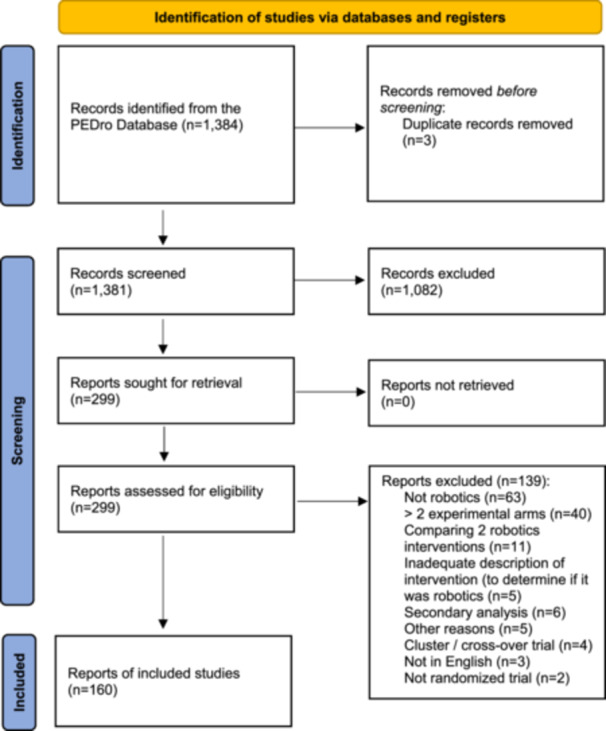
Flow of studies through the review.

We retrieved 1384 records in PEDro, screened 1381 titles and abstracts, and examined 299 full texts. After examining full texts, 160 trials were included. Figure 1 shows the flow of studies through the screening process.

### 3.2 Characteristics of Included Trials

Trials were mainly performed with neurological populations (95%) and were from Europe (41.3%), Asia (37.5%), and North America (17.9%) (Table 1). The mean (SD) PEDro score was 6.0 (1.4) and ranged from 2 to 8, with 61% of trials being of high quality. All trials were randomized and no trials blinded participants or therapists. Concealed allocation (41.9%) and intention‐to‐treat analysis (35.6%) were the items that were least met. Most trials defined primary outcomes (72.5%) or timepoints (63.5%), had 1 (21.9%), 2–5 (40.0%), or 6–10 (20.0%) primary outcomes and 1 (60.6%) or 2 (26.9%) primary timepoints. Less than half of trials (45.0%) had registered protocols. Abstract word counts were mainly 176–225 (23.8%), 226–275 (35.0%), and 276–325 (20.6%) words.

**Table 1:** Characteristics of the included trials (*n* = 160).

Characteristics	% or mean (SD)
Patient population	
Neurological	95
Orthopaedics	3.1
Burns	1.9
Geographical location of trial	
Europe	41.3
Asia	37.5
North America	16.9
Other (Africa, South America, Oceania)	4.4
Publication year	
≤ 2010	8.8
2011–2015	27.5
2016–2020	38.1
2021–2024	25.6
Primary outcomes	
Defined/undefined	72.5/27.5
Number of primary outcomes^a^	
1	21.9
2–5	40
6–10	20
11–15	10.6
16+	7.5
Primary timepoints	
Defined/undefined	64.4/35.6
Number of primary timepoints^b^	
1	60.6
2	26.9
3+	12.5
Registered protocol	45.0
Abstract word count	
≤ 175	6.3
176–225	23.8
226–275	35.0
276–325	20.6
≥ 326	14.4
Quality assessment (% Yes)	
Eligibility criteria (not scored)	81.9
Randomisation	100.0
Concealed allocation	41.9
Baseline similarities	93.1
Subject blinding	0.0
Therapist blinding	0.0
Assessor blinding	68.1
Adequate follow‐up	64.4
Intention‐to‐treat analysis	35.6
Between‐group comparisons	97.5
Point estimates and variability	94.3
Total PEDro score	6.0 (1.4)

a If trials did not nominate a primary outcome all outcomes were deemed primary. If trials only assessed 1 outcome measure that outcome measure was deemed primary.

^b^ If trials did not nominate a primary timepoint all measurement timepoints were deemed primary. If trials only assessed 1 timepoint (excluding baseline) that timepoint was deemed primary.

### 3.3 Assessment of Spin

The most common form of spin was failing to mention adverse events (90.0%) followed by overenthusiastic interpretation of nonsignificant primary outcomes as effective (76.9%) (Table 2). For spin item 3, most trials (96.3%) did not have any negative primary outcomes and were scored not relevant. The amount of positive spin items in each trial ranged from 0 to 6; 0 items (6 trials, 3.1%), 1 item (25 trials, 15.6%), 2 items (34 trials, 21.3%), 3 items (17 trials, 10.6%), 4 items (37 trials, 23.1%), 5 items (29 trials, 18.1%), 6 items (13 trials, 8.1%), and 7 items (0 trials, 0%).

**Table 2:** Number of trials that scored Yes (spin present), No (spin not present) and Not relevant for each spin item.

Spin item	Yes (spin present) *n* (%)	No (spin not present) *n* (%)	Not relevant *n* (%)
1. Omission of primary outcomes	78^a^ (48.8)	82 (51.3)	N/A
2. Fail to report non‐significant primary outcomes	84^a^ (52.5)	50 (31.3)	26 (16.3)
3. Selective reporting of positive results and omission of negative results of primary outcomes	2 (1.3)	4 (2.5)	154 (96.3)
4. Focus on statistically significant outcomes other than the primary	48 (30.0)	65 (40.6)	47 (29.4)
5. Fail to mention adverse events of the intervention	144 (90.0)	16 (10.0)	N/A
6. Overenthusiastic interpretation of statistically nonsignificant primary outcomes results as effective	123 (76.9)	10 (6.3)	27 (16.9)
7. Recommendation of a treatment without a clinically important effect on the primary outcome	36 (22.5)	1 (0.6)	123 (76.9)

Abbreviations: *n* = number of trials; N/A = not applicable.

a For spin item 1 and 2, some trials reported the primary outcome but only reported within group differences. Assuming the trial had reported within group differences of all primary outcomes, we stated they had not omitted primary outcomes (spin item 1) but had failed to report nonsignificant primary outcomes (spin 2) as most of the between group differences were nonsignificant.

### 3.4 Rater Agreement

Percent agreement between raters ranged from 72.5% to 95.6%. Fleiss' *κ* values were moderate (items 4 and 5), weak (items 1 and 2), minimal (items 6 and 7) and slight agreement that was no better than chance (item 3) (Table 3).

**Table 3:** Fleiss' *κ* values and percent agreement between raters for each spin item.

Spin item	Fleiss' *κ* (95% CI's)	Percent agreement (%)
1. Omission of primary outcomes	0.48 (0.32–0.63)	75.0
2. Fail to report nonsignificant primary outcomes	0.53 (0.37–0.68)	76.3
3. Selective reporting of positive results and omission of negative results of primary outcomes	0.10 (−0.05 to 0.26)	92.5
4. Focus on statistically significant outcomes other than the primary	0.60 (0.45–0.76)	83.1
5. Fail to mention adverse events of the intervention	0.78 (0.62–0.93)	95.6
6. Overenthusiastic interpretation of statistically nonsignificant primary outcomes results as effective	0.35 (0.20–0.51)	72.5
7. Recommendation of a treatment without a clinically important effect on the primary outcome	0.28 (0.12–0.43)	73.8

### 3.5 Exploratory Analysis

One‐way ANOVA showed no significant between group differences for geographical location (*F*_(3, 156)_ = 0.45, *p* = 0.99), abstract word count (*F*_(4, 155)_ = 1.32, *p* = 0.26), journal 2‐year citation/document (*F*_(4, 155)_ = 0.63, *p* = 0.64), whether the trial was funded (*F*_(2, 157)_ = 0.74, *p* = 0.49), whether the trial had a COI (*F*_(2, 157)_ = 0.87, *p* = 0.42), whether the trial had an industry COI (*F*_(2, 157)_ = 0.29, *p* = 0.75). Unpaired *t*‐tests showed no difference in the amount of spin between trials with and without a registered protocol (MD: 0.39, 95% CI: −0.14 to 0.91), with or without a defined primary outcome (MD: 0.145, 95% CI −0.44 to 0.73) and between trials in neurological physiotherapy and Other areas (MD: 0.23, 95% CI: −0.97 to 1.42). Regression analyses showed that the amount of spin was not associated with year of publication (unstandardized *β*: −0.02, 95% CI −0.08 to 0.04). The amount of spin was significantly associated with trial quality (unstandardized *β*: −0.22, 95% CI −0.41 to −0.03), with higher quality trials having lower spin.

## 4 Discussion

The current study found spin in most abstracts of physiotherapy trials investigating robotics with 97% of trials containing at least one item of spin. Most commonly, authors failed to report adverse events and provided an overenthusiastic interpretation of nonsignificant results. Approximately half of the trials focussed on the significant results of their primary outcomes and failed to report non‐significant primary outcomes. Over one‐fifth of trials recommended a treatment without a clinically important effect on the primary outcome, however most did not make any clinical recommendation. Exploratory analyses showed that the amount of spin in abstracts was weakly related to trial quality and not related to abstract word count, geographical location, protocol registration, publication year, COI or funding.

### 4.1 Comparison With Other Literature

Non‐reporting of adverse events was the most common example of abstract spin. This is comparable with trials in physiotherapy for low back pain that also found that non‐reporting of adverse events was high (93.5%) [6]. Adverse events are frequently underreported in non‐medical intervention trials [47]. Nonreporting of the adverse events in the abstract (and often the full‐text) in robotics trials could due to minor nature of the adverse events experienced. As adverse events are poorly reported, it is difficult gauge the impact of the adverse events experienced although conceivably these could include minor events such as skin irritation, redness, abrasions, skin break down, fear of falling, motion sickness and general discomfort or pain. Moderate events could include falls, muscle tears, shoulder subluxation. Although the events may have been minor, or not happened at all, these should be reported in the abstract so readers can make an informed treatment decision or provide a patient with an informed choice to use or not use the intervention. Even if minor, adverse events should still be reported in the abstract or a statement that no adverse events occurred is needed, as information on the safety of an intervention is crucial for the implementation of the intervention. Providing this in the abstract provides transparency which is why reporting of adverse events is recommended in the Consolidated Standards of Reporting Trials (CONSORT) statement [80, 81]. Although the authors intention may not be to mislead the reader, it is important to report such issues to ensure transparency for all consumers of the literature.

Overenthusiastic interpretation of statistically non‐significant primary outcomes was another commonly scored item. In comparison to previous literature, this was more common in robotic trials than in trials in low back pain (61.5%) [6], traumatic brain injury (60.6%) [38] and physiotherapy (73%) [37]. This was mainly due to conclusions on the effectiveness of interventions being based on within‐group changes, when between‐group differences were nonsignificant. This is not surprising as many reviews have highlighted that robotic interventions have indifferent or small treatment effects. Although the intentions of the authors cannot be assumed, using within‐group comparisons makes the robotics interventions appear effective when they are not. This overstates the effectiveness of the interventions. It is important that these trials report between‐group comparisons to ensure that placebo effects, Hawthorne effects, regression to the mean and natural recovery are controlled for. As such, interpreting treatment effectiveness from within‐group comparisons is inappropriate [82] and demonstrates poor statistical/methodological rigour.

Reporting of any negative findings (i.e., favoring the control over the robotic intervention) was limited, with only 2/6 trials with negative findings reporting negative findings in the abstract and 154 trials not reporting any negative findings. Such an overwhelmingly positive slant could suggest selective reporting of positive outcomes or selective publication of trials with positive results [83, 84]. Although assessing the content and accuracy of protocols were beyond the scope of this study, 55% of trials did not refer to any protocol. This means that outcomes could be selected or changed, without any *a priori* record, as is commonly done in physiotherapy literature [85, 86]. As a result, negative outcomes in robotic literature are likely more common but the extent of this remains unknown.

More than one in five robotics trials recommended a treatment without a clinically meaningful effect of the primary outcome. This is concerning as many trials based their recommendation of treatment on statistical significance rather than clinical significance. This is not unique to robotics trials, but the potential implications and equipment required for people wishing to use the interventions, compared to other physiotherapy interventions, are more costly. If presented appropriately, the interventions will appear less effective or ineffective, and may not be used further. Further, readers may have unrealistic expectations of treatment effectiveness which may result in unjustified optimism of the treatment for the clinician and patient. In 2022, the International Society of Physiotherapy Editors endorsed the use of an estimation‐based approach [87, 88] as well as reporting effect sizes and 95% CI relative to the smallest clinically worthwhile effect. As most of the trials in the current review were published prior to 2022 and many articles were published in journals that were not part of the joint editorial, it is unrealistic to expect that any of the trials will have followed this advice. However, it would help the interpretation of the clinical results in abstracts. To aid in the interpretation of trials relative to the SCWE, authors are encouraged to use guidelines proposed by Kamper [89] or Herbert et al. [90].

The exploratory analyses showed that most investigated factors did not explain the amount of spin in a trial. Some trials investigating the relationship between factors such as journal impact factor [91], trial quality [6], abstract word count [6], trial registration [32], multicentre trials [6], and amount of international collaboration [32] have shown significant relationships while others have shown no relationships for any variable [4, 7, 92]. The only significant exploratory test was that spin was associated with trial quality. The effect was very small as for a 7‐point increase in spin (the scale range), the PEDro quality would reduce by 1.61 points. Although studies, including ours, have shown that that high spin is related to total lower trial quality [6], these findings are not universal [93].

### 4.2 User Agreement of the Spin Checklist With Recommendations

Kappa and agreement values using previous versions of this checklist have been minimal to weak [6]. We created and updated item definitions and performed five calibrations of abstract spin ratings on diverse trials prior to rating abstracts in an attempt to improve user agreement. However, the kappa values remained minimal or weak (4 items) or no better than chance (1 item). A reason for poor agreement was likely due to the difficulty in identifying the primary outcome/timepoint when studies did not define a primary outcome/timepoint. If the primary outcome/timepoints were identified incorrectly, spin items 1–4, 6, and 7 could be scored incorrectly. Seventy‐three percent of studies did not report primary outcomes, and 64% of studies did not report primary timepoints (when there was more than one outcome/timepoint). Some studies, such as those investigating gait parameters had 20+ outcomes, without defining a primary outcome. We would recommend adjusting the checklist so that if the primary outcome is not defined, then the using the first mentioned outcome in the methods section. In studies with more than 1 timepoint, when a primary timepoint has not been defined, then the timepoint immediately after the intervention could be used. These changes would reduce the disagreements because of the misidentification of primary outcomes.

For spin item 3 “…omission of negative results of primary outcomes” raters sometimes confused “negative” results and “non‐significant” results. For this item, having a highlighted explanation of what “negative” means within the checklist would be helpful. It is also important to ensure that practice trials are purposely selected so that at least one practice trial reports a negative result.

The direction of a significantly “better” outcome measure was sometimes unclear or misinterpreted (i.e., higher scores were considered better, when lower scores were better). This was particularly problematic for studies on gait parameters or studies with unfamiliar outcome measures. Ideally, prior to assessing articles for spin, authors could have an “outcome measures” spreadsheet, with the direction of positive findings. This can be added to if a new outcome measure is encountered.

For spin item 6, the overenthusiastic interpretation of statistically nonsignificant items as effective, could have been broken into its multiple parts such as “using with group differences” and “using verbiage to imply significance.” This would provide a more discriminative assessment of this item.

For spin item 7, ambiguous recommendations were challenging to score with authors using vague, positively slanted language such as “…seems promising in gait rehabilitation…may be useful to plan highly patient‐tailored gait rehabilitation protocols…” [94] or “…is a treatment option….” [95]. This ambiguity in recommendations was frequent and resulted in disagreements between raters. Having strict a priori defined rules about what constitutes a recommendation, including multiple examples would improve agreement.

Further, guidance documents should be created. These documents should account for more scenarios and provide examples for these scenarios.

### 4.3 Strengths and Limitations

The strength of our study is that we assessed spin in all physiotherapy related trials in robotics. The spin checklist was refined and trialled using multiple raters. Each rater was provided five calibration trials to ensure familiarity with the spin checklist. Although there were some deviations from protocol, these have been transparently reported and justified.

There were also some limitations. We were perhaps overly stringent in applying the spin checklist. For example, many trials did not specify the primary timepoint, used multiple analyses (i.e., between group analyses and regression analyses), or analyzed the items of a scale instead of the total scale. In these instances, the abstracts needed to report the results of all timepoints/analyses or provide an appropriate summary to be scored as having no spin. The overreliance on the primary outcome in the current checklist is potentially problematic especially when 28% of trials did not define a primary outcome. In trials that didn't report a primary outcome, all outcomes were deemed primary. This meant that these trials had many primary outcomes, and it would be very difficult to report all outcomes in the abstract. Sometimes in the same trial, some primary analyses were (clinically) significant and some were non‐significant, yet a positive clinical recommendation was made. These were deemed as having spin, although this could be seen as being overly stringent. Ideally, we would like authors to identify their primary outcomes and comment on the uncertainty of findings for the results of primary outcomes. We only included two‐armed trials and excluded multiarmed trials. Our rationale was to ensure that the trials were as comparable as possible. Trials with more arms would have more primary comparisons which may increase the likelihood of spin given the limited space in the abstract. We only searched the PEDro database which could be seen as a limitation as we may have potential missed trials. Despite this, the PEDro database is a comprehensive overview of physiotherapy literature and has significant overlap of indexed physiotherapy trials with CENTRAL, EMBASE and PubMed [96]. The PEDro database indexes trials on physiotherapy interventions or interventions that could become physiotherapy interventions in the future, regardless of registration status, journal, language, or methodological quality [49, 50]. Further, PEDro is updated with monthly searches of Medline, Embase, CINAHL, CENTRAL AMED, and PsycINFO with citation tracking and notification of new studies from individuals (such as academics or clinicians) [50, 51]. Although the PEDro scale has an “advanced search option,” it lacks Medical Subject Headings or the ability to search using complex search strings which may limit search sensitivity.

### 4.4 Future Directions

Ninety‐seven percent of studies in robotics articles contain spin. This means that many robotics abstracts are misrepresenting the articles that they are summarizing. This is concerning as the treatments are being misrepresented which impacts reader perception of treatment effectiveness and could determine if people access a study. There needs to be more discussion of abstract spin in robotics, and its potential consequences, through discussions in conferences, editorials, letters, online forums and education of all article stakeholders. Given the cost of many robotic interventions, it is important that these are presented fairly in the abstract.

Currently, there is no accepted spin checklist for monitoring abstract spin. Our study has contributed to a checklist by trialing a modified checklist and providing suggestions for a future checklist. Standardization of an abstract spin checklist would aid in the comparability between studies investigating abstract spin, and an endorsement by CONSORT or the International Society of Physiotherapy Editors would increase the visibility of abstract spin in the literature. A uniform and widely accepted spin checklist would be useful for authors who write abstracts, journal editors who are the gatekeepers to articles being published and consumers who can read an abstract and identify spin. Given this, eliminating spin or ensuring that people are educated about spin is important to reduce the potential impacts of spin.

## 5 Conclusion

Ninety‐seven percent physiotherapy trials investigating robotic interventions contained spin. We caution consumers of robotics research to engage thoroughly and critically with articles in this field of literature. Most commonly authors failed to report adverse events and overenthusiastically interpreted statistically non‐significant primary outcomes. The inter‐rater agreement of the checklist ranged from slight agreement that was no better than chance to moderate agreement. We would recommend further refining the definitions of each checklist item.

## Author Contributions

**Hilary Tier:** investigation, writing – review and editing. **Jana Verveer:** investigation, writing – review and editing. **David B. Anderson:** investigation, writing – review and editing. **Camila Quel De Oliveira:** investigation, writing – review and editing. **Nicci Bartley:** investigation, writing – review and editing, methodology. **Poonam Mehta:** conceptualization, investigation, writing – review and editing, methodology. **Rafael Z. Pinto:** conceptualization, investigation, methodology, writing – review and editing. **Arianne P. Verhagen:** conceptualization, investigation, writing – review and editing, methodology. **Alana B. McCambridge:** conceptualization, investigation, writing – review and editing, methodology. **Peter W. Stubbs:** conceptualization, investigation, writing – original draft, visualization, validation, writing – review and editing, project administration, supervision, formal analysis, methodology.

## Funding

The authors received no specific funding for this work.

## Ethics Statement

The authors have nothing to report.

## Consent

The authors have nothing to report.

## Conflicts of Interest

The authors declare no conflicts of interest.

## Supporting information

Supplmentary dataset SUBMIT.

Declaration+of+Interest FORM.

## Data Availability

The data that supports the findings of this study are available in the Supporting Information of this article. Data are provided as supporting files.

## Appendix 1

Included studies

1. E. M. Abd El‐Kafy, M. A. Alshehri, A. A. R. El‐Fiky, M. A. Guermazi, and H. M. Mahmoud, “The Effect of Robot‐Mediated Virtual Reality Gaming on Upper Limb Spasticity Poststroke: A Randomized‐Controlled Trial,” *Games for Health Journal* 11, no. 2 (2022): 93–103, http://10.1089/g4h.2021.0197.
2. H. A. Abdullah, C. Tarry, C. Lambert, S. Barreca, and B. O. Allen, “Results of Clinicians Using a Therapeutic Robotic System in an Inpatient Stroke Rehabilitation Unit,” *Journal of NeuroEngineering and Rehabilitation* 8, no. 1 (2011): 50, http://10.1186/1743-0003-8-50.
3. M. Alcobendas‐Maestro, A. Esclarín‐Ruz, R. M. Casado‐López, et al., “Lokomat Robotic‐Assisted Versus Overground Training Within 3 to 6 Months of Incomplete Spinal Cord Lesion: Randomized Controlled Trial,” *Neurorehabilitation & Neural Repair* 26, no. 9 (2012): 1058–1063, http://10.1177/1545968312448232.
4. J. F. Alingh, B. M. Fleerkotte, B. E. Groen, et al., “Effect of Assist‐as‐Needed Robotic Gait Training on the Gait Pattern Poststroke: A Randomized Controlled Trial,” *J NeuroEngineering Rehabil* 2021;18(1):26, http://10.1186/s12984-020-00800-4.
5. G. J. Androwis, B. M. Sandroff, P. Niewrzol, et al., “A Pilot Randomized Controlled Trial of Robotic Exoskeleton‐Assisted Exercise Rehabilitation in Multiple Sclerosis,” *Multiple Sclerosis and Related Disorders* 2021;51:102936, http://10.1016/j.msard.2021.102936.
6. D. H. Bangand and W. S. Shin, “Effects of Robot‐Assisted Gait Training on Spatiotemporal Gait Parameters and Balance in Patients With Chronic Stroke: A Randomized Controlled Pilot Trial,” *NRE* 2016;38(4):343–349, http://10.3233/NRE-161325.
7. O. Bayındır, G. Akyüz, and N. Sekban, “The Effect of Adding Robot‐Assisted Hand Rehabilitation to Conventional Rehabilitation Program Following Stroke: A Randomized‐Controlled Study,” *Turk J Phys Med Rehab* 2022;68(2):254–261, http://10.5606/tftrd.2022.8705.
8. S. Beer, B. Aschbacher, D. Manoglou, E. Gamper, J. Kool, and J. Kesselring, “Robot‐Assisted Gait Training in Multiple Sclerosis: A Pilot Randomized Trial,” *Mult Scler* 2008;14(2):231–236, http://10.1177/1352458507082358.
9. J. Bergmann, C. Krewer, K. Jahn, F. Müller, “Robot‐Assisted Gait Training to Reduce Pusher Behavior: A Randomized Controlled Trial,” *Neurology* 2018;91(14), http://10.1212/WNL.0000000000006276.
10. R. Berriozabalgoitia, I. Bidaurrazaga‐Letona, E. Otxoa, M. Urquiza, J. Irazusta, and A. Rodriguez‐Larrad, “Overground Robotic Program Preserves Gait in Individuals With Multiple Sclerosis and Moderate to Severe Impairments: A Randomized Controlled Trial,” *Archives of Physical Medicine and Rehabilitation*. 2021;102(5):932–939, http://10.1016/j.apmr.2020.12.002.
11. L. Cai, Y. Liu, Z. Wei, H. Liang, Y. Liu, and M. Cui, “Robot‐Assisted Rehabilitation Training Improves Knee Function and Daily Activity Ability in Older Adults Following Total Knee Arthroplasty,” *Research in Nursing & Health* 2023;46(2):203–209, http://10.1002/nur.22290.
12. R. S. Calabrò, A. Naro, and M. Russo, et al., “Shaping Neuroplasticity by Using Powered Exoskeletons in Patients With Stroke: A Randomized Clinical Trial,” *J NeuroEngineering Rehabil* 2018;15(1):35, http://10.1186/s12984-018-0377-8.
13. R. S. Calabrò, M. Accorinti, B. Porcari, et al., “Does Hand Robotic Rehabilitation Improve Motor Function by Rebalancing Interhemispheric Connectivity After Chronic Stroke? Encouraging Data From a Randomized‐Clinical‐Trial,” *Clinical Neurophysiology* 2019;130(5):767–780, http://10.1016/j.clinph.2019.02.013.
14. M. Capecci, S. Pournajaf, D. Galafate, et al., “Clinical Effects of Robot‐Assisted Gait Training and Treadmill Training for Parkinson's Disease. A Randomized Controlled Trial,” *Annals of Physical and Rehabilitation Medicine* 2019;62:303–312, http://10.1016/j.rehab.2019.06.016.
15. S. Carda, M. Invernizzi, A. Baricich, C. Comi, A. Croquelois, and C. Cisari. “Robotic Gait Training Is not Superior to Conventional Treadmill Training in Parkinson Disease: A Single‐Blind Randomized Controlled Trial,” *Neurorehabil Neural Repair* 2012;26(9):1027–1034, http://10.1177/1545968312446753.
16. I. Carpinella, T. Lencioni, T. Bowman, et al., “Effects of Robot Therapy on Upper Body Kinematics and Arm Function in Persons Poststroke: A Pilot Randomized Controlled Trial,” *J NeuroEngineering Rehabil* 2020;17:10, http://10.1186/s12984-020-0646-1.
17. W. H. Chang, M. S. Kim, J. P. Huh, P. K. W. Lee, and Y. H. Kim. “Effects of Robot‐Assisted Gait Training on Cardiopulmonary Fitness in Subacute Stroke Patients: A Randomized Controlled Study,” *Neurorehabil Neural Repair* 2012;26(4):318–324, http://10.1177/1545968311408916.
18. S. H. Chang and T. Afzal, “TIRR SCI Clinical Exoskeleton Group, Berliner J, and Francisco G. Exoskeleton‐Assisted Gait Training to Improve Gait in Individuals With Spinal Cord Injury: A Pilot Randomized Study,” *Pilot Feasibility Stud* 2018;4:62, http://10.1186/s40814-018-0247-y.
19. Z. J. Chen, C. He, F. Guo, C. H. Xiong, and X. L. Huang, “Exoskeleton‐Assisted Anthropomorphic Movement Training (EAMT) for Poststroke Upper Limb Rehabilitation: A Pilot Randomized Controlled Trial,” *Archives of Physical Medicine and Rehabilitation* 2021;102(11):2074–2082, http://1010.1016/j.apmr.2021.06.001.
20. E. Y. Y. Cheung, K. K. K. Yu, R. L. C. Kwan, C. K. M. Ng, R. M. W. Chau, and G. L. Y. Cheing, “Effect of EMG‐Biofeedback Robotic‐Assisted Body Weight Supported Treadmill Training on Walking Ability and Cardiopulmonary Function on People With Subacute Spinal Cord Injuries—A Randomized Controlled Trial,” *BMC Neurol* 2019;19:140, http://10.1186/s12883-019-1361-z.
21. Y. S. Cho, S. Y. Joo, and C. H. Seo, “Effect of Robot‐Assisted Gait Training on the Biomechanical Properties of Burn Scars: A Single‐Blind, Randomized Controlled Trial,” *Burns & Trauma* 2022;10:tkac026, http://10.1093/burnst/tkac026.
22. Y. S. Choi, K. W. Lee, J. H. Lee, S. B. Kim, G. T. Park, and S. J. Lee, “The Effect of an Upper Limb Rehabilitation Robot on Hemispatial Neglect in Stroke Patients,” *Ann Rehabil Med* 2016;40(4):611–619, http://10.5535/arm.2016.40.4.611.
23. J. Chua, J. Culpan, and E. Menon, “Efficacy of an Electromechanical Gait Trainer Poststroke in Singapore: A Randomized Controlled Trial,” *Archives of Physical Medicine and Rehabilitation* 2016;97:683–690, http://10.1016/j.apmr.2015.12.025.
24. Ç. Çinar, M. A. Yildirim, K. Öneş, and G. Gökşenoğlu, “Effect of Robotic‐Assisted Gait Training on Functional Status, Walking and Quality of Life in Complete Spinal Cord Injury,” *International Journal of Rehabilitation Research* 2021;44:262–268, http://10.1097/MRR.0000000000000486.
25. I. Clerici, D. Ferrazzoli, R. Maestri, et al., “Rehabilitation in Progressive Supranuclear Palsy: Effectiveness of Two Multidisciplinary Treatments,” *PLoS ONE* 2017;12(2):e0170927, http://10.1371/journal.pone.0170927.
26. J. J. Daly, N. Hogan, E. M. Perepezko, et al., “Response to Upper‐Limb Robotics and Functional Neuromuscular Stimulation Following Stroke,” *JRRD* 2005;42(6):723–736, http://10.1682/JRRD.2005.02.0048.
27. K. Daunoraviciene, A. Adomaviciene, A. Grigonyte, J. Griškevičius, and A. Juocevicius, “Effects of Robot‐Assisted Training on Upper Limb Functional Recovery During the Rehabilitation of Poststroke Patients,” *THC* 2018;26:S533–S542, http://10.3233/THC-182500.
28. R. C. de Araújo, F. L. Junior, D. N. Rocha, T. S. Sono, and M. Pinotti, “Effects of Intensive Arm Training With an Electromechanical Orthosis in Chronic Stroke Patients: A Preliminary Study,” *Archives of Physical Medicine and Rehabilitation* 2011;92:1746–1753, http://10.1016/j.apmr.2011.05.021.
29. R. De Luca, G. Maresca, T. Balletta, et al., “Does Overground Robotic Gait Training Improve Non‐Motor Outcomes in Patients With Chronic Stroke? Findings From a Pilot Study,” *Journal of Clinical Neuroscience* 2020;81:240–245, http://10.1016/j.jocn.2020.09.070.
30. S. Dehem, M. Gilliaux, G. Stoquart, et al., “Effectiveness of Upper‐Limb Robotic‐Assisted Therapy in the Early Rehabilitation Phase After Stroke: A Single‐Blind, Randomized, Controlled Trial,” *Annals of Physical and Rehabilitation Medicine* 2019;62:313–320, http://10.1016/j.rehab.2019.04.002.
31. M. Druzbicki, W. Rusek, M. Szczepanik, J. Dudek, and A. Snela, “Assessment of the Impact of Orthotic Gait Training on Balance in Children With Cerebral Palsy,” *Acta of Bioengineering and Biomechanics* 2010;12(2):53–58.
32. M. Drużbicki, W. Rusek, S. Snela, et al., “Functional Effects of Robotic‐Assisted Locomotor Treadmill Thearapy in Children With Cerebral Palsy,” *J Rehabil Med* 2013;45:358–363, http://10.2340/16501977-1114.
33. S. M. El‐Shamy, “Efficacy of Armeo Robotic Therapy Versus Conventional Therapy on Upper Limb Function in Children With Hemiplegic Cerebral Palsy,” *Am J Phys Med Rehabil* 2018;97:164–169, http://10.1097/PHM.0000000000000852.
34. A. Esquenazi, S. Lee, A. T. Packel, and L. Braitman, “A Randomized Comparative Study of Manually Assisted Versus Robotic‐Assisted Body Weight Supported Treadmill Training in Persons With a Traumatic Brain Injury,” *PM&R* 2013;5:280–290, http://10.1016/j.pmrj.2012.10.009.
35. R. W. Evans, C. L. Shackleton, S. West, et al., “Robotic Locomotor Training Leads to Cardiovascular Changes in Individuals With Incomplete Spinal Cord Injury Over a 24‐Week Rehabilitation Period: A Randomized Controlled Pilot Study,” *Archives of Physical Medicine and Rehabilitation* 2021;102:1447–1456, http://10.1016/j.apmr.2021.03.018.
36. P. Feys, K. Coninx, L. Kerkhofs, et al., “Robot‐Supported Upper Limb Training in a Virtual Learning Environment: A Pilot Randomized Controlled Trial in Persons With MS,” *J NeuroEngineering Rehabil* 2015;12:60, http://10.1186/s12984-015-0043-3.
37. S. Fisher, L. Lucas, and T. A. Thrasher, “Robot‐Assisted Gait Training for Patients With Hemiparesis Due to Stroke,” *Topics in Stroke Rehabilitation* 2011;18(3):269–276, http://10.1310/tsr1803-269.
38. L. W. Forrester, A. Roy, A. Krywonis, G. Kehs, H. I. Krebs, and R. F. Macko, “Modular Ankle Robotics Training in Early Subacute Stroke: A Randomized Controlled Pilot Study,” *Neurorehabil Neural Repair* 2014;28(7):678–687, http://10.1177/1545968314521004.
39. M. Franceschini, S. Mazzoleni, M. Goffredo, et al., “Upper Limb Robot‐Assisted Rehabilitation Versus Physical Therapy on Subacute Stroke Patients: A Follow‐Up Study,” *Journal of Bodywork and Movement Therapies* 2020;24:194–198, http://10.1016/j.jbmt.2019.03.016.
40. A. Frisoli, M. Barsotti, E. Sotgiu, G. Lamola, C. Procopio, and C. Chisari, “A Randomized Clinical Control Study on the Efficacy of Three‐Dimensional Upper Limb Robotic Exoskeleton Training in Chronic Stroke,” *J NeuroEngineering Rehabil* 2022;19:14, http://10.1186/s12984-022-00991-y.
41. A. Furnari, R. S. Calabrò, M. C. De Cola, et al., “Robotic‐Assisted Gait Training in Parkinson's Disease: A 3‐Month Follow‐Up Randomized Clinical Trial,” *International Journal of Neuroscience* 2017;127(11):996–1004, http://10.1080/00207454.2017.1288623.
42. M. Galli, V. Cimolin, M. De Pandis, et al., “Robot‐Assisted Gait Training Versus Treadmill Training in Patients With Parkinson's Disease: A Kinematic Evaluation With Gait Profile Score,” *FN* 2016;31(3):163–170, http://10.11138/FNeur/2016.31.3.163.
43. M. Gandolfi, C. Geroin, A. Picelli, et al., “Robot‐Assisted Versus Sensory Integration Training in Treating Gait and Balance Dysfunctions in Patients With Multiple Sclerosis: A Randomized Controlled Trial,” *Front Hum Neurosci* 2014;8:318, http://10.3389/fnhum.2014.00318.
44. M. Gandolfi, N. Valè, E. Dimitrova, et al., “Robot‐Assisted Stair Climbing Training on Postural Control and Sensory Integration Processes in Chronic Post‐Stroke Patients: A Randomized Controlled Clinical Trial,” *Front Neurosc*i 2019;13:1143, http://10.3389/fnins.2019.01143.
45. M. Gilliaux, A. Renders, D. Dispa, et al., “Upper Limb Robot‐Assisted Therapy in Cerebral Palsy: A Single‐Blind Randomized Controlled Trial,” *Neurorehabil Neural Repair* 2015;29(2):183–192, http://10.1177/1545968314541172.
46. P. H. Gorman, W. Scott, L. VanHiel, K. E. Tansey, W. M. Sweatman, and P. R. Geigle, “Comparison of Peak Oxygen Consumption Response to Aquatic and Robotic Therapy in Individuals With Chronic Motor Incomplete Spinal Cord Injury: A Randomized Controlled Trial,” *Spinal Cord* 2019;57:471–481, http://10.1038/s41393-019-0239-7.
47. A. Güç, M. A. Çebiçci, S. T. Sütbeyaz, H. T. Çalış, and H. Abakay, “Comparison of the Effectiveness of Conventional Physiotherapy Methods and Robot‐Assisted Gait Training After Botulinum Toxin Injection of Lower Extremities in Children With Cerebral Palsy: Prospective Randomized Controlled Study,” *Journal of Pediatric Neurology* 2024;22:194–201, http://10.1055/s-0043-1769737.
48. E. Y. Han, S.H. Im, B. R. Kim, M. J. Seo, and M. O. Kim, “Robot‐Assisted Gait Training Improves Brachial–Ankle Pulse Wave Velocity and Peak Aerobic Capacity in Subacute Stroke Patients With Totally Dependent Ambulation: Randomized Controlled Trial,” *Medicine* 2016;95:41, http://10.1097/MD.0000000000005078.
49. S. Hesse, C. Werner, M. Pohl, S. Rueckriem, J. Mehrholz, and M. L. Lingnau, “Computerized Arm Training Improves the Motor Control of the Severely Affected Arm After Stroke: A Single‐Blinded Randomized Trial in Two Centers,” *Stroke* 2005;36:1960–1966, http://10.1161/01.STR.0000177865.37334.ce.
50. S. Hesse, A. Heß, C. C. Werner, N. Kabbert, and R. Buschfort, “Effect on Arm Function and Cost of Robot‐Assisted Group Therapy in Subacute Patients With Stroke and a Moderately to Severely Affected Arm: A Randomized Controlled Trial,” *Clin Rehabil*. 2014;28(7):637–647, http://10.1177/0269215513516967.
51. J. Hidler, D. Nichols, M. Pelliccio, et al., “Multicenter Randomized Clinical Trial Evaluating the Effectiveness of the Lokomat in Subacute Stroke,” *Neurorehabil Neural Repair* 2009;23(1):5–13, http://10.1177/1545968308326632.
52. T. G. Hornby, D. D. Campbell, J. H. Kahn, T. Demott, J. L. Moore, and H. R. Roth, “Enhanced Gait‐Related Improvements After Therapist‐ Versus Robotic‐Assisted Locomotor Training in Subjects With Chronic Stroke: A Randomized Controlled Study,” *Stroke* 2008;39:1786–1792, http://10.1161/STROKEAHA.107.504779.
53. Y. Hsieh wei, C. Wu yi, W. Wang en, et al., “Bilateral Robotic Priming Before Task‐Oriented Approach in Subacute Stroke Rehabilitation: A Pilot Randomized Controlled Trial,” *Clin Rehabil* 2017;31(2):225–233, http://10.1177/0269215516633275.
54. H. Hsu, H. Chiu, T. Kuan, C. Tsai, F. Su, L. Kuo, “Robotic‐Assisted Therapy With Bilateral Practice Improves Task and Motor Performance in the Upper Extremities of Chronic Stroke Patients: A Randomized Controlled Trial,” *Aus Occup Therapy J* 2019;66:637–647, http://10.1111/1440-1630.12602.
55. C. Hu, X. Wang, and T. Pan, “Effect of Acupuncture Combined With Lower Limb Gait Rehabilitation Robot on Improving Walking Function in Stroke Patients With Hemiplegia,” *NRE* 2024;54:309–317, http://10.3233/NRE-230258.
56. B. Husemann, F. Müller, C. Krewer, S. Heller, and E. Koenig, “Effects of Locomotion Training With Assistance of a Robot‐Driven Gait Orthosis in Hemiparetic Patients After Stroke: A Randomized Controlled Pilot Study,” *Stroke* 2007;38:349–354, http://10.1161/01.STR.0000254607.48765.cb.
57. K. Jiae, M. H. Chun, J. Lee, J. W. Kim, and J. Y. Lee, “Intensity Control of Robot‐Assisted Gait Training Based on Biometric Data: Preliminary Study,” *Medicine* 2022;101(38):e30818, http://10.1097/MD.0000000000030818.
58. S. Y. Joo, S. Y. Lee, Y. S. Cho, K. J. Lee, and C. H. Seo, “Effects of Robot‐Assisted Gait Training in Patients With Burn Injury on Lower Extremity: A Single‐Blind, Randomized Controlled Trial,” *JCM* 2020;9:2813, http://10.3390/jcm9092813.
59. C. Jung, D. Y. Kim, S. Kwon, M. H. Chun, J. Kim, and S. H. Kim, “Morning Walk‐Assisted Gait Training Improves Walking Ability and Balance in Patients With Ataxia: A Randomized Controlled Trial,” *Brain Neurorehabil* 2020;13(3):e23, http://10.12786/bn.2020.13.e23.
60. L. E. Kahn, M. L. Zygman, W. Z. Rymer, and D. J. Reinkensmeyer, “Robot‐Assisted Reaching Exercise Promotes Arm Movement Recovery in Chronic Hemiparetic Stroke: A Randomized Controlled Pilot Study,” *J NeuroEngineering Rehabil* 2006;3:12, http://10.1186/1743-0003-3-12.
61. C. J. Kang, M. H. Chun, J. Lee, and J. Y. Lee, “Effects of Robot (SUBAR)‐Assisted Gait Training in Patients With Chronic Stroke: Randomized Controlled Trial,” *Medicine* 2021;100(48):e27974, http://10.1097/MD.0000000000027974.
62. C. P. Kelley, J. Childress, C. Boake, and EA. Noser, “Over‐Ground and Robotic‐Assisted Locomotor Training in Adults With Chronic Stroke: A Blinded Randomized Clinical Trial,” *Disability and Rehabilitation: Assistive Technolog*y 2013;8(2):161–168, http://10.3109/17483107.2012.714052.
63. S. Y. Kim, L. Yang, I. J. Park, et al., “Effects of Innovative WALKBOT Robotic‐Assisted Locomotor Training on Balance and Gait Recovery in Hemiparetic Stroke: A Prospective, Randomized, Experimenter Blinded Case Control Study With a 4‐Week Follow‐Up,” *IEEE Trans Neural Syst Rehabil Eng* 2015;23(4):636–642, http://10.1109/TNSRE.2015.2404936.
64. J. Kim, D. Y. Kim, M. H. Chun, et al., “Effects of Robot‐(Morning Walk) Assisted Gait Training for Patients After Stroke: A Randomized Controlled Trial,” *Clin Rehabil* 2019;33(3):516–523, http://10.1177/0269215518806563.
65. S. H. Kim, D. M. Ji, I. S. Hwang, et al., “Three‐Dimensional Magnetic Rehabilitation, Robot‐Enhanced Hand‐Motor Recovery after Subacute Stroke: A Randomized Controlled Trial,” *Brain Sciences* 2023;13(12):1685, http://10.3390/brainsci13121685.
66. V. Klamroth‐Marganska, J. Blanco, K. Campen, et al., “Three‐Dimensional, Task‐Specific Robot Therapy of the Arm After Stroke: A Multicentre, Parallel‐Group Randomized Trial,” *Lancet Neurology* 2014;13(2):159–166, http://10.1016/S1474-4422(13)70305-3.
67. S. Klobucká, R. Klobucký, and B. Kollár, “Effect of Robot‐Assisted Gait Training on Motor Functions in Adolescent and Young Adult Patients With Bilateral Spastic Cerebral Palsy: A randomized controlled trial,” *NRE* 2020;47(4):495–508, http://10.3233/NRE-203102.
68. S. V. Kotov, E. V. Isakova, V. Lijdvoy Yu, et al., “Robotic Restoration of Gait Function in Patients in the Early Recovery Period of Stroke,” *Neurosci Behav Physi* 2021;51(5):583–589, http://10.1007/s11055-021-01109-y.
69. S. V. Kotov, A. RomanovI, E. V. Silina, et al., “Efficiency of Leg Exoskeleton Use in Rehabilitation of Cerebral Stroke Patients,” *Serbian Journal of Experimental and Clinical Research* 2021;22(3):257–264, http://10.2478/sjecr-2021-0045.
70. I. Kröger, C. Nerz, L. Schwickert, et al., “Robot‐Assisted Training After Proximal Humeral Fracture: A Randomized Controlled Multicentre Intervention Trial,” *Clin Rehabil* 2021;35(2):242–252, http://10.1177/0269215520961654.
71. N. G. Kutner, R. Zhang, A. J. Butler, S. L. Wolf, and J. L. Alberts, “Quality‐of‐Life Change Associated With Robotic‐Assisted Therapy to Improve Hand Motor Function in Patients With Subacute Stroke: A Randomized Clinical Trial,” *Physical Therapy* 2010;90(4):493–504, http://10.2522/ptj.20090160.
72. K. W. Lee, S. B. Kim, J. H. Lee, S. J. Lee, and S. W. Yoo, “Effect of Upper Extremity Robot‐Assisted Exercise on Spasticity in Stroke Patients,” *Ann Rehabil Med* 2016;40(6):961, http://10.5535/arm.2016.40.6.961.
73. M. J. Lee, J. H. Lee, and S. M. Lee, “Effects of Robot‐Assisted Therapy on Upper Extremity Function and Activities of Daily Living in Hemiplegic Patients: A Single‐Blinded, Randomized, Controlled Trial,” *THC* 2018;26(4):659–666, http://10.3233/THC-181336.
74. J. Lee, D. Y. Kim, S. H. Lee, et al., “End‐Effector Lower Limb Robot‐Assisted Gait Training Effects in Subacute Stroke Patients: A Randomized Controlled Pilot Trial,” *Medicine* 2023;102(42):e35568, http://10.1097/MD.0000000000035568.
75. R. J. M. Lemmens, A. A. A. Timmermans, Y. J. M. Janssen‐Potten, et al., “Accelerometry Measuring the Outcome of Robot‐Supported Upper Limb Training in Chronic Stroke: A Randomized Controlled Trial,” *PLoS ONE* 2014;9(5):e96414, http://10.1371/journal.pone.0096414.
76. J. Li, T. Wu, Z. Xu, and X. Gu, “A Pilot Study of Post‐Total Knee Replacement Gait Rehabilitation Using Lower Limbs Robot‐Assisted Training System,” *Eur J Orthop Surg Traumatol* 2014;24(2):203–208, http://10.1007/s00590-012-1159-9.
77. W. Liao wen, W. Chang ying, Y. Hsieh wei, K. Lin chung, and W. Chang ying, “Effects of Robot‐Assisted Upper Limb Rehabilitation on Daily Function and Real‐World Arm Activity in Patients With Chronic Stroke: A Randomized Controlled Trial,” *Clin Rehabil* 2012;26(2):111–120, http://10.1177/0269215511416383.
78. S. M. Linder, A. B. Rosenfeldt, R. C. Bay, K. Sahu, S. L. Wolf, and J. L. Alberts, “Improving Quality of Life and Depression After Stroke Through Telerehabilitation,” *American Journal of Occupational Therapy* 2015;69(2):6902290020p1–6902290020p10, http://10.5014/ajot.2015.014498.
79. D. R. Louie, W. B. Mortenson, M. Durocher, et al., “Efficacy of an Exoskeleton‐Based Physical Therapy Program for Non‐Ambulatory Patients During Subacute Stroke Rehabilitation: A Randomized Controlled Trial,” *J NeuroEngineering Rehabil* 2021;18(1):149, http://10.1186/s12984-021-00942-z.
80. T. T. Ma, Q. Zhang, T. T. Zhou, et al., “Effects of Robotic‐Assisted Gait Training on Motor Function and Walking Ability in Children With Thoracolumbar Incomplete Spinal Cord Injury,” *NRE* 2022;51(3):499–508, http://10.3233/NRE-220124.
81. M. G. Maggio, A. Naro, R. De Luca, et al., “Body Representation in Patients With Severe Spinal Cord Injury: A Pilot Study on the Promising Role of Powered Exoskeleton for Gait Training,” *JPM* 2022;12(4):619, http://10.3390/jpm12040619.
82. S. Masiero, A. Celia, M. Armani, and G. Rosati, “A Novel Robot Device in Rehabilitation of Post‐Stroke Hemiplegic Upper Limbs,” *Aging Clin Exp Res* 2006;18(6):531–535, http://10.1007/BF03324854.
83. S. Masiero, M. Armani, and G. Rosati, “Upper‐Limb Robot‐Assisted Therapy in Rehabilitation of Acute Stroke Patients: Focused Review and Results of New Randomized Controlled Trial,” *JRRD* 2011;48(4):355, http://10.1682/JRRD.2010.04.0063.
84. S. Masiero, M. Armani, G. Ferlini, G. Rosati, and A. Rossi, “Randomized Trial of a Robotic Assistive Device for the Upper Extremity During Early Inpatient Stroke Rehabilitation,” *Neurorehabil Neural Repair* 2014;28(4):377–386, http://10.1177/1545968313513073.
85. A. Mayr, E. Quirbach, A. Picelli, M. Kofler, N. Smania, and L. Saltuari, “Early Robot‐Assisted Gait Retraining in Non‐Ambulatory Patients With Stroke: A Single Blind Randomized Controlled Trial,” *Eur J Phys Rehabil Med* 2019;54(6):819–826, http://10.23736/S1973-9087.18.04832-3.
86. M. Mıdık, N. Paker, D. Buğdaycı, and A. Mıdık, “Effects of Robot‐Assisted Gait Training on Lower Extremity Strength, Functional Independence, and Walking Function in Men With Incomplete Traumatic Spinal Cord Injury,” *Turk J Phys Med Rehab* 2020;66(1):54–59, http://10.5606/tftrd.2020.3316.
87. F. Moll, A. Kessel, A. Bonetto, et al., “Use of Robot‐Assisted Gait Training in Pediatric Patients With Cerebral Palsy in an Inpatient Setting—A Randomized Controlled Trial,” *Sensors* 2022;22(24):9946, http://10.3390/s22249946.
88. Y. G. Nam, J. W. Lee, J. W. Park, et al., “Effects of Electromechanical Exoskeleton‐Assisted Gait Training on Walking Ability of Stroke Patients: A Randomized Controlled Trial,” *Archives of Physical Medicine and Rehabilitation* 2019;100(1):26–31, http://10.1016/j.apmr.2018.06.020.
89. Y. Nam, J. Park, H. Lee, et al., “Effects of Electromechanically Assisted Gait Trainer With Exowalk in Patients With Chronic Stroke: A Randomized Controlled Trial,” *J Rehabil Med* 2020;52:jrm00097, http://10.2340/16501977-2723.
90. Y. G. Nam, M. J. Ko, S. K. Bok, et al., “Efficacy of Electromechanical‐Assisted Gait Training on Clinical Walking Function and Gait Symmetry After Brain Injury of Stroke: A Randomized Controlled Trial.” *Sci Rep* 2022;12(1):6880, http://10.1038/s41598-022-10889-3.
91. X. Niu, D. Varoqui, M. Kindig, and M. M. Mirbagheri, “Prediction of Gait Recovery in Spinal Cord Injured Individuals Trained With Robotic Gait Orthosis,” *J NeuroEngineering Rehabil* 2014;11(1):42, http://10.1186/1743-0003-11-42.
92. M. Ochi, F. Wada, S. Saeki, and K. Hachisuka, “Gait Training in Subacute Non‐Ambulatory Stroke Patients Using a Full Weight‐bEaring Gait‐Assistance Robot: A Prospective, Randomized, Open, Blinded‐Endpoint Trial,” *Journal of the Neurological Sciences* 2015;353(1–2):130–136, http://10.1016/j.jns.2015.04.033.
93. T. Ogino, Y. Kanata, R. Uegaki, et al., “Effects of Gait Exercise Assist Robot (GEAR) on Subjects With Chronic Stroke: A Randomized Controlled Pilot Trial,” *Journal of Stroke and Cerebrovascular Diseases* 2020;29(8):104886, http://10.1016/j.jstrokecerebrovasdis.2020.104886.
94. T. Ogino, Y. Kanata, R. Uegaki, et al., “Improving Abnormal Gait Patterns by Using a Gait Exercise Assist Robot (GEAR) in Chronic Stroke Subjects: A Randomized, Controlled, Pilot Trial,” *Gait & Posture* 2020;82:45–51, http://10.1016/j.gaitpost.2020.07.017.
95. F. Orihuela‐Espina, G. F. Roldán, I. Sánchez‐Villavicencio, et al., “Robot Training for Hand Motor Recovery in Subacute Stroke Patients: A Randomized Controlled Trial,” *Journal of Hand Therapy* 2016;29(1):51–57, http://10.1016/j.jht.2015.11.006.
96. T. Ozsoy‐Unubol, E. Ata, M. Cavlak, S. Demir, Z. Candan, and F. Yilmaz, “Effects of Robot‐Assisted Gait Training in Patients With Multiple Sclerosis: A Single‐Blinded Randomized Controlled Study,” *Am J Phys Med Rehabil* 2022;101(8):768–774, http://10.1097/PHM.0000000000001913.
97. S. J. Page, V. Hill, and S. White, “Portable Upper Extremity Robotics Is as Efficacious as Upper Extremity Rehabilitative Therapy: A Randomized Controlled Pilot Trial,” *Clin Rehabil* 2013;27(6):494–503, http://10.1177/0269215512464795.
98. .C Park, M. Oh‐Park, A .Bialek, K. Friel, D. Edwards, and J. S. H. You, “Abnormal Synergistic Gait Mitigation in Acute Stroke Using an Innovative Ankle–Knee–Hip Interlimb Humanoid Robot: A Preliminary Randomized Controlled Trial,” *Sci Rep* 2021;11(1):22823, http://10.1038/s41598-021-01959-z.
99. A. Picelli, C. Melotti, F. Origano, et al., “Robot‐Assisted Gait Training in Patients With Parkinson Disease: A Randomized Controlled Trial,” *Neurorehabil Neural Repair* 2012;26(4):353–361, http://10.1177/1545968311424417.
100. A. Picelli, C. Melotti, F. Origano, A. Waldner, R. Gimigliano, and N. Smania, “Does Robotic Gait Training Improve Balance in Parkinson's Disease? A Randomized Controlled Trial,” *Parkinsonism & Related Disorders* 2012;18(8):990–993, http://10.1016/j.parkreldis.2012.05.010.
101. A. Picelli, C. Melotti, F. Origano, et al., “Robot‐Assisted Gait Training Is Not Superior to Balance Training for Improving Postural Instability in Patients With Mild to Moderate Parkinson's Disease: A Single‐Blind Randomized Controlled Trial,” *Clin Rehabil* 2015;29(4):339–347, http://10.1177/0269215514544041.
102. A. Picelli, M. Bacciga, C. Melotti, et al., “Combined Effects of Robot‑Assisted Gait Training and Botulinum Toxin Type A on Spastic Equinus Foot in Patients With Chronic Stroke: A Pilot, Single Blind, Randomized Controlled Trial,” *European Journal of Physical and Rehabilitation Medicine* 2016;52(6):759–766.
103. A. Picelli, D. Munari, A. Modenese, et al., “Robot‐Assisted Arm Training for Treating Adult Patients With Distal Radius Fracture: A Proof‐Of‐Concept Pilot Study,” *Eur J Phys Rehabil Med* 2020;56(4), http://10.23736/S1973-9087.20.06112-2.
104. A. Piira, A. Lannem, M. Sørensen, et al., “Robot‐Assisted Locomotor Training Did Not Improve Walking Function in Patients With Chronic Incomplete Spinal Cord Injury: A Randomized Clinical Trial,” *J Rehabil Med* 2019;51(5):385–389, http://10.2340/16501977-2547.
105. M. Pohl, C. Werner, M. Holzgraefe, et al., “Repetitive Locomotor Training and Physiotherapy Improve Walking and Basic Activities of Daily Living After Stroke: A Single‐Blind, Randomized Multicentre Trial (DEutsche GAngtrainerStudie, DEGAS),” *Clin Rehabil* 2007;21(1):17–27, http://10.1177/0269215506071281.
106. A. Pompa, G. Morone, M. Iosa, et al., “Does Robot‐Assisted Gait Training Improve Ambulation in Highly Disabled Multiple Sclerosis People? A Pilot Randomized Control Trial,” *Mult Scler* 2017;23(5):696–703, http://10.1177/1352458516663033.
107. Q. Qian, X. Hu, Q. Lai, S. C. Ng, Y. Zheng, and W. Poon, “Early Stroke Rehabilitation of the Upper Limb Assisted With an Electromyography‐Driven Neuromuscular Electrical Stimulation‐Robotic Arm,” *Front Neurol* 2017;8:447, http://10.3389/fneur.2017.00447.
108. L. Raciti, L. Pignolo, V. Perini, et al., “Improving Upper Extremity Bradykinesia in Parkinson's Disease: A Randomized Clinical Trial on the Use of Gravity‐Supporting Exoskeletons,” *JCM* 2022;11(9):2543, http://10.3390/jcm11092543.
109. D. J. Reinkensmeyer, E. T. Wolbrecht, V. Chan, C. Chou, S. C. Cramer, and J. E. Bobrow, “Comparison of 3D, Assist‐as‐Needed Robotic Arm/Hand Movement Training Provided with Pneu‐WREX to Conventional Table Top Therapy Following Chronic Stroke,” *American Journal of Physical Medicine & Rehabilitation* 2013;91:S232–S241.
110. O. Rémy‐Néris, A. Le Jeannic, A. Dion, et al., “Additional, Mechanized Upper Limb Self‐Rehabilitation in Patients With Subacute Stroke: The REM‐AVC Randomized Trial,” *Stroke* 2021;52(6):1938–1947, http://10.1161/STROKEAHA.120.032545.
111. M. Russo, V. Dattola, M. C. De Cola, et al., “The Role of Robotic Gait Training Coupled With Virtual Reality in Boosting the Rehabilitative Outcomes in Patients With Multiple Sclerosis,” *International Journal of Rehabilitation Research* 2018;41(2):166–172, http://10.1097/MRR.0000000000000270.
112. P. Sale, M. F. De Pandis, D. Le Pera, et al., “Robot‐Assisted Walking Training for Individuals With Parkinson's Disease: A Pilot Randomized Controlled Trial,” *BMC Neurol* 2013;13(1):50, http://10.1186/1471-2377-13-50.
113. P. Sale, M. Franceschini, S. Mazzoleni, E. Palma, M. Agosti, and F. Posteraro, “Effects of Upper Limb Robot‐Assisted Therapy on Motor Recovery in Subacute Stroke Patients,” *J NeuroEngineering Rehabil* 2014;11(1):104, http://10.1186/1743-0003-11-104.
114. P. Sale, S. Mazzoleni, V. Lombardi, et al, “Recovery of Hand Function With Robot‐Assisted Therapy in Acute Stroke Patients: A Randomized‐Controlled Trial,” *International Journal of Rehabilitation Research* 2014;37(3):236–242, http://10.1097/MRR.0000000000000059.
115. A. F. Samhan, N. M. Abdelhalim, and R. K. Elnaggar, “Effects of Interactive Robot‐Enhanced Hand Rehabilitation in Treatment of Paediatric Hand‐burns: A Randomized, Controlled Trial With 3‐Months Follow‐Up,” *Burns* 2020;46(6):1347–1355, http://10.1016/j.burns.2020.01.015.
116. R. S. M. Sarhan, M. F. Chevidikunnan, and R. A. M. Gaowgzeh, “Locomotor Treadmill Traning Program Using Driven Gait Orthosis Versus Manual Treadmill Therapy on Motor Output in Spastic Diplegic Cerebral Palsy Children,” *Journal of Health and Allied Sciences NU* 2014;4(4):10–17, http://10.1055/s-0040-1703824.
117. I. Schwartz, A. Sajin, I. Fisher, et al., “The Effectiveness of Locomotor Therapy Using Robotic‐Assisted Gait Training in Subacute Stroke Patients: A Randomized Controlled Trial,” *PM&R* 2009;1(6):516–523, http://10.1016/j.pmrj.2009.03.009.
118. I. Schwartz, A. Sajin, E. Moreh, et al., “Robot‐Assisted Gait Training in Multiple Sclerosis Patients: A Randomized Trial,” *Mult Scler* 2012;18(6):881–890, http://10.1177/1352458511431075.
119. R. R. Serrezuela, M. T. Quezada, M. H. Zayas, A. M. Pedrón, D. M. Hermosilla, and R. S. Zamora, “Robotic Therapy for the Hemiplegic Shoulder Pain: A Pilot Study,” *J NeuroEngineering Rehabil* 2020;17(1):54, http://10.1186/s12984-020-00674-6.
120. D. Setoguchi, K. Kinoshita, S. Kamada, et al., “Hybrid Assistive Limb Improves Restricted Hip Extension After Total Hip Arthroplasty,” *Assistive Technology* 2022;34(1):112–120, http://10.1080/10400435.2020.1712498.
121. A. A. A. Sherief, A. S. Abdelfattah, and M. S. Elfakharany, “Electrodiagnostic effect of Armeo Robotic Therapy versus Conventional Therapy in Erb's Palsy Children,” *Ann Clin Anal Med* 2021;12(Suppl_01):35–40, http://10.4328/ACAM.20324.
122. J. C. Shin, J. Y. Kim, H. K. Park, and N. Y. Kim, “Effect of Robotic‐Assisted Gait Training in Patients With Incomplete Spinal Cord Injury,” *Ann Rehabil Med* 2014;38(6):719, http://10.5535/arm.2014.38.6.719.
123. J. C. Shin, H. R. Jeon, D. Kim, et al., “Effects of End‐Effector Robot‐Assisted Gait Training on Gait Ability, Muscle Strength, and Balance in Patients With Spinal Cord Injury,” *NRE* 2023;53(3):335–346, http://10.3233/NRE-230085.
124. N. Smania, P. Bonetti, M. Gandolfi, et al., “Improved Gait After Repetitive Locomotor Training in Children With Cerebral Palsy,” *American Journal of Physical Medicine & Rehabilitation* 2011;90(2):137–149, http://10.1097/PHM.0b013e318201741e.
125. K. J. Song, M. H. Chun, J. Lee, and C. Lee, “The Effect of Robot‐Assisted Gait Training on Cortical Activation in Stroke Patients: A Functional Near‐Infrared Spectroscopy Study,” *NRE* 2021;49(1):65–73, http://10.3233/NRE-210034.
126. J. Stein, L. Bishop, D. J. Stein, and C. K. Wong, “Gait Training With a Robotic Leg Brace After Stroke: A Randomized Controlled Pilot Study,” *American Journal of Physical Medicine & Rehabilitation* 2014;93(11):987–994, http://10.1097/PHM.0000000000000119.
127. R. Stolz, R. Nayyar, J. Louie, K. J. Bower, S. K. Paul, and L. Ng, “The Effectiveness of a Novel Cable‐Driven Gait Trainer (Robowalk) Combined With Conventional Physiotherapy Compared to Conventional Physiotherapy Alone Following Stroke: A Randomized Controlled Trial,” *International Journal of Rehabilitation Research* 2019;42(4):377–384, http://10.1097/MRR.0000000000000375.
128. S. Straudi, M. G. Benedetti, E. Venturini, M. Manca, C. Foti, and N. Basaglia, “Does Robot‐Assisted Gait Training Ameliorate Gait Abnormalities in Multiple Sclerosis? A Pilot Randomized‐Control Trial,” *NRE* 2013;33(4):555–563, http://10.3233/NRE-130990.
129. S. Straudi, C. Fanciullacci, C. Martinuzzi, et al., “The Effects of Robot‐Assisted Gait Training in Progressive Multiple Sclerosis: A Randomized Controlled Trial,” *Mult Scler* 2016;22(3):373–384, http://10.1177/1352458515620933.
130. S. Straudi, F. Manfredini, N. Lamberti, C. Martinuzzi, E. Maietti, and N. Basaglia, “Robot‐Assisted Gait Training is Not Superior to Intensive Overground Walking in Multiple Sclerosis With Severe Disability (The RAGTIME Study): A Randomized Controlled Trial,” *Mult Scler* 2020;26(6):716–724, http://10.1177/1352458519833901.
131. K. Takahashi, K. Domen, T. Sakamoto, et al., “Efficacy of Upper Extremity Robotic Therapy in Subacute Poststroke Hemiplegia: An Exploratory Randomized Trial,” *Stroke* 2016;47(5):1385–1388, http://10.1161/STROKEAHA.115.012520.
132. M. Talaty and A. Esquenazi, “Feasibility and Outcomes of Supplemental Gait Training by Robotic and Conventional Means in Acute Stroke Rehabilitation,” *J NeuroEngineering Rehabil* 2023;20(1):134, http://10.1186/s12984-023-01243-3.
133. G. Taveggia, A. Borboni, C. Mulé, J. H. Villafañe, and S. Negrini, “Conflicting Results of Robot‐Assisted Versus Usual Gait Training During Postacute Rehabilitation of Stroke Patients: A Randomized Clinical Trial,” *International Journal of Rehabilitation Research* 2016;39(1):29–35, http://10.1097/MRR.0000000000000137.
134. G. Taveggia, A. Borboni, L. Salvi, et al., “Efficacy of Robot‐Assisted Rehabilitation for the Functional Recovery of the Upper Limb in Post‐Stroke Patients: A Randomized Controlled Study,” *European Journal of Physical and Rehabilitation Medicine* 2016;52(6):767–773.
135. N. Thimabut, P. Yotnuengnit, J. Charoenlimprasert, et al., “Effects of the Robot‐Assisted Gait Training Device Plus Physiotherapy in Improving Ambulatory Functions in Patients With Subacute Stroke With Hemiplegia: An Assessor‐Blinded, Randomized Controlled Trial,” *Archives of Physical Medicine and Rehabilitation* 2022;103(5):843–850, http://10.1016/j.apmr.2022.01.146.
136. A. A. Timmermans, R. J. Lemmens, M. Monfrance, et al., “Effects of Task‐Oriented Robot Training on Arm Function, Activity, and Quality of Life in Chronic Stroke Patients: A Randomized Controlled Trial,” *J NeuroEngineering Rehabil* 2014;11(1):45, http://10.1186/1743-0003-11-45.
137. T. J. D. Tomić, A. M. Savić, A. S. Vidaković, et al., “ArmAssist Robotic System Versus Matched Conventional Therapy for Poststroke Upper Limb Rehabilitation: A Randomized Clinical Trial,” *BioMed Research International* 2017;2017:1–7, http://10.1155/2017/7659893.
138. K. Tomida, S. Sonoda, S. Hirano, et al., “Randomized Controlled Trial of Gait Training Using Gait Exercise Assist Robot (GEAR) in Stroke Patients With Hemiplegia,” *Journal of Stroke and Cerebrovascular Diseases* 2019;28(9):2421–2428, http://10.1016/j.jstrokecerebrovasdis.2019.06.030.
139. D. Uçar, N. Paker, and D. Buğdaycı, “Lokomat: A Therapeutic Chance for Patients With Chronic Hemiplegia,” *NRE* 2014;34(3):447–453, http://10.3233/NRE-141054.
140. M. P. M. Van Nunen, K. H. L. Gerrits, M. Konijnenbelt, T. W. J. Janssen, and A. De Haan, “Recovery of Walking Ability Using a Robotic Device in Subacute Stroke Patients: A Randomized Controlled Study,” *Disability and Rehabilitation: Assistive Technology* 2015;10(2):141‐148, http://10.3109/17483107.2013.873489.
141. C. Vaney, B. Gattlen, V. Lugon‐Moulin, et al., “Robotic‐Assisted Step Training (Lokomat) Not Superior to Equal Intensity of Over‐Ground Rehabilitation in Patients With Multiple Sclerosis,” *Neurorehabil Neural Repair* 2012;26(3):212–221, http://10.1177/1545968311425923.
142. B. T. Volpe, D. Lynch, A. Rykman‐Berland, et al., “Intensive Sensorimotor Arm Training Mediated by Therapist or Robot Improves Hemiparesis in Patients With Chronic Stroke,” *Neurorehabil Neural Repair* 2008;22(3):305–310, http://10.1177/1545968307311102.
143. A. Wall, J. Borg, and S. Palmcrantz, “Self‐Perceived Functioning and Disability After Randomized Conventional and Electromechanically‐Assisted Gait Training in Subacute Stroke: A 6 Months Follow‐Up,” *NRE* 2019;45(4):501–511, http://10.3233/NRE-192929.
144. L. Wallard, G. Dietrich, Y. Kerlirzin, and J. Bredin, “Robotic‐Assisted Gait Training Improves Walking Abilities in Diplegic Children With Cerebral Palsy,” *European Journal of Paediatric Neurology* 2017;21(3):557–564, http://10.1016/j.ejpn.2017.01.012.
145. L. Wallard, G. Dietrich, Y. Kerlirzin, and J. Bredin, “Effect of Robotic‐Assisted Gait Rehabilitation on Dynamic Equilibrium Control in the Gait of Children With Cerebral Palsy,” *Gait & Posture* 2018;60:55–60, http://10.1016/j.gaitpost.2017.11.007.
146. K. P. Westlake and C. Patten, “Pilot Study of Lokomat Versus Manual‐Assisted Treadmill Training for Locomotor Recovery Post‐Stroke,” *J NeuroEngineering Rehabil* 2009;6(1):18, http://10.1186/1743-0003-6-18.
147. S. L. Wolf, K. Sahu, R. C. Bay, et al, “The HAAPI (Home Arm Assistance Progression Initiative) Trial: A Novel Robotics Delivery Approach in Stroke Rehabilitation,” *Neurorehabil Neural Repair* 2015;29(10):958–968, http://10.1177/1545968315575612.
148. A. Wright, K. Stone, L. Martinelli, et al., “Effect of Combined Home‐Based, Overground Robotic‐Assisted Gait Training and Usual Physiotherapy on Clinical Functional Outcomes in People With Chronic Stroke: A Randomized Controlled Trial,” *Clin Rehabil* 2021;35(6):882–893, http://10.1177/0269215520984133.
149. M. Wu, J. Kim, P. Arora, D. J. Gaebler‐Spira, and Y. Zhang., “Effects of the Integration of Dynamic Weight Shifting Training Into Treadmill Training on Walking Function of Children with Cerebral Palsy: A Randomized Controlled Study,” *American Journal of Physical Medicine & Rehabilitation* 2017;96(11):765–772, http://10.1097/PHM.0000000000000776.
150. M. Wu, J. Kim, and F. Wei, “Facilitating Weight Shifting During Treadmill Training Improves Walking Function in Humans With Spinal Cord Injury: A Randomized Controlled Pilot Study,” *Am J Phys Med Rehabil* 2018;97(8):585–592, http://10.1097/PHM.0000000000000927.
151. X. N. Xiang, H. Y. Zong, Y. Ou, et al., “Exoskeleton‐Assisted Walking Improves Pulmonary Function and Walking Parameters Among Individuals With Spinal Cord Injury: A Randomized Controlled Pilot Study,” *J NeuroEngineering Rehabil* 2021;18(1):86, http://10.1186/s12984-021-00880-w.
152. X. N. Xiang, L. M. Zhang, H. Y. Zong, et al., “Exoskeleton‐Assisted Walking for Pulmonary and Exercise Performances of SCI Individuals,” *IEEE Trans Neural Syst Rehabil Eng* 2023;31:39–47, http://10.1109/TNSRE.2022.3215652.
153. B. Yaşar, E. Atıcı, D. A. Razaei, and T. Ç. Saldıran, “Effectiveness of Robot‐Assisted Gait Training on Functional Skills in Children With Cerebral Palsy,” *Journal of Pediatric Neurology* 2022;20(03):164–170, http://10.1055/s-0041-1725128.
154. L. F. Yeung, C. Ockenfeld, M. K. Pang, et al., “Randomized Controlled Trial of Robot‐Assisted Gait Training With Dorsiflexion Assistance on Chronic Stroke Patients Wearing Ankle‐Foot‐Orthosis,” *J NeuroEngineering Rehabil* 2018;15(1):51, http://10.1186/s12984-018-0394-7.
155. C. Yokota, K. Tanaka, K. Omae, et al, “Effect of Cyborg‐Type Robot Hybrid Assistive Limb on Patients With Severe Walking Disability in Acute Stroke: A Randomized Controlled Study,” *Journal of Stroke and Cerebrovascular Diseases* 2023;32(4):107020, http://10.1016/j.jstrokecerebrovasdis.2023.107020.
156. D. H. Yoo, Y. J. Cha, S. K. Kim, and J. S. Lee, “Effect of Three‐Dimensional Robot‐Assisted Therapy on Upper Limb Function of Patients With Stroke,” *J Phys Ther Sci* 2013;25(4):407–409, 10.1589/jpts.25.407.
157. M. Yoo, M. H. Chun, G. R. Hong, C. Lee, J. K. Lee, and A. Lee, “Effects of Training With a Powered Exoskeleton on Cortical Activity Modulation in Hemiparetic Chronic Stroke Patients: A Randomized Controlled Pilot Trial,” *Archives of Physical Medicine and Rehabilitation* 2023;104(10):1620–1629, http://10.1016/j.apmr.2023.05.012.
158. D. Yu, Z. Yang, L. Lei, N. Chaoming, and W. Ming, “Robot‐Assisted Gait Training Plan for Patients in Poststroke Recovery Period: A Single Blind Randomized Controlled Trial,” Zhou P, ed. *BioMed Research International* 2021;2021:1–7, http://10.1155/2021/5820304.
159. N. Yun, M. C. Joo, S. C. Kim, and M. S. Kim, “Robot‐Assisted Gait Training Effectively Improved Lateropulsion in Subacute Stroke Patients: A Single‐Blinded Randomized Controlled Trial,” *Eur J Phys Rehabil Med* 2019;54(6), http://10.23736/S1973-9087.18.05077-3.
160. H. Zhang, X. Li, Y. Gong, et al., “Three‐Dimensional Gait Analysis and sEMG Measures for Robotic‐Assisted Gait Training in Subacute Stroke: A Randomized Controlled Trial,” *BioMed Research International* 2023, no. 1 (2023): 7563802, http://10.1155/2023/7563802.

## Appendix 2

Spin checklist. Modified checklist published with permission (Stubbs et al. (2020) *Phys Ther Rev*. 26:102–108. http://10.1080/10833196.2020.1832708)

Spin item 1: Omission of primary outcomes

- Score Yes if
  ∘ the results of 1 primary outcome (either presented as within or between group differences) have been omitted
- Score No if
  ∘ the results of all primary outcomes are mentioned
  *Spin item 2: Fail to report statistically nonsignificant primary outcomes*
- Score Yes if
  ∘ the results of the between group differences of 1 nonsignificant primary outcomes have been omitted
- Score No if
  ∘ all between‐group differences for significant and nonsignificant primary outcomes have been included
  ∘ the results of between‐group differences of negative primary outcomes have been omitted, but all nonsignificant items have been reported
- Score Not Relevant if
  ∘ there are no statistically between‐group differences for nonsignificant primary outcomes
  *Spin item 3: Selective reporting of positive results and omission of negative results of primary outcomes*
- Score Yes if
  ∘ The results of the between group differences of 1 negative primary outcome(s) have been omitted and there is 1 positive primary outcome(s)
- Score No if
  ∘ the results of the between group differences of 1 negative primary outcome(s) have been omitted and there are no positive primary outcome(s)
  ∘ the results of all the between group differences of negative primary outcomes have been included
- Score Not Relevant if
  ∘ there are no negative between group differences of primary outcomes

*Spin item 4: Focus on statistically significant outcomes other than the primary*

- Score Yes if
  ∘ 1 significant secondary outcome is reported, and 1 primary between‐group outcome is not reported (regardless of being positive, negative or indifferent).
  ∘ The a between group primary outcome is not reported and statistically significant subgroup analyses are included or groups are modified without a priori statement (i.e., grouping responders and nonresponders)
- Score No if
  ∘ All between‐group primary outcomes are reported
- Score Not Relevant if
  ∘ no primary outcomes are defined (and all outcomes are deemed primary)
  *Spin item 5: Fail to mention adverse events*
- Score Yes if
  ∘ the abstract does not report adverse events (whether they occurred or not)
  ∘ results in the full‐text mentions minor or major adverse events and the abstract reports that there were “no adverse events”
  ∘ results in the full‐text do not mention adverse events and the abstract does not mention adverse events
  ∘ the full‐text reports no adverse events, and the abstract does not report that there were “no adverse events”
- Score No if
  ∘ the abstract reports “adverse events occurring” or “adverse events not occurring,” and this is consistent with the full‐text
  *Spin item 6: Overenthusiastic interpretation of statistically non‐significant primary outcomes results as effective*
- Score Yes if
  ∘ the benefit or effectiveness of an intervention are interpreted based on within group (pre vs. post) statistically significant differences only rather than between group differences
  ∘ data that was nonsignificant are interepreted as though it was a statistically significant positive result, including but not limited to phrases such as “trend towards” or “approaching significance”
- Score No if
  ∘ nonsignificant primary outcomes are interpreted appropriately
- Score Not Relevant if
  ∘ there are no nonsignificant primary outcomes
  *Spin item 7: Recommendation of a treatment without a clinically important effect on primary outcomes*
- Score Yes if
  ∘ 95% confidence intervals includes the smallest clinically worthwhile effect (SCWE)
  ∘ Treatment is recommended due to “safety” and “tolerability,” and not due to a SCWE in the primary outcome (unless safety/tolerability is explicitly stated as being the primary outcome compared/assessed a priori)
- Score No if
  ∘ clinical importance or meaningfulness is defined a‐priori and the results of the trial support a clinically important change
- Score Not Relevant if
  ∘ clinical importance is not reported in the abstract

Notes

- In cases where no primary outcomes are defined, all outcomes are deemed primary
- Trials will be assumed to be inequality trials unless non‐inferiority or equivalence (or synonyms) are explicitly stated in the aims or methods. In such cases, statistically nonsignificant results can result in the recommendation of the treatment, so long as the comparator is included in the interpretation, and so long as the comparator is clinically effective.
- If clinical importance or meaningfulness is not defined a‐priori we developed an arbitrary rule that the SCWE was 15% of the scale range when a scale was used or a 15% difference between measures of the post‐intervention value of the intervention group for continuous measures
